# Molecular Dynamics Simulation Reveals Correlated Inter-Lobe Motion in Protein Lysine Methyltransferase SMYD2

**DOI:** 10.1371/journal.pone.0145758

**Published:** 2015-12-30

**Authors:** Nicholas Spellmon, Xiaonan Sun, Nualpun Sirinupong, Brian Edwards, Chunying Li, Zhe Yang

**Affiliations:** 1 Department of Biochemistry and Molecular Biology, Wayne State University School of Medicine, Detroit, Michigan, United States of America; 2 Nutraceuticals and Functional Food Research and Development Center, Prince of Songkla University, Hat-Yai, Songkhla, Thailand; University of Maryland, UNITED STATES

## Abstract

SMYD proteins are an exciting field of study as they are linked to many types of cancer-related pathways. Cardiac and skeletal muscle development and function also depend on SMYD proteins opening a possible avenue for cardiac-related treatment. Previous crystal structure studies have revealed that this special class of protein lysine methyltransferases have a bilobal structure, and an open–closed motion may regulate substrate specificity. Here we use the molecular dynamics simulation to investigate the still-poorly-understood SMYD2 dynamics. Cross-correlation analysis reveals that SMYD2 exhibits a negative correlated inter-lobe motion. Principle component analysis suggests that this correlated dynamic is contributed to by a twisting motion of the C-lobe with respect to the N-lobe and a clamshell-like motion between the lobes. Dynamical network analysis defines possible allosteric paths for the correlated dynamics. There are nine communities in the dynamical network with six in the N-lobe and three in the C-lobe, and the communication between the lobes is mediated by a lobe-bridging β hairpin. This study provides insight into the dynamical nature of SMYD2 and could facilitate better understanding of SMYD2 substrate specificity.

## Introduction

SMYD is a special class of protein lysine methyltransferases involved in heart and muscle development [1, 2]. SMYD linked to tumorigenesis opens a possible avenue for cancer treatment [2, 3]. SMYD proteins contain five members, SMYD1–5 [2, 4–7]. Each member contains a conserved SET domain that is “split” by a zinc-finger MYND domain [2]. The SET domain is a catalytic unit responsible for protein lysine methylation [8]. The MYND domain is a protein–protein interaction module and has also been shown to have a DNA binding ability in SMYD proteins [3, 9, 10]. Among SMYD proteins, SMYD2 has the broadest substrate specificity. In addition to histone proteins, SMYD2 is able to methylate p53, retinoblastoma tumor suppressor (RB), estrogen receptor α (ERα), poly(ADP-ribose) polymerase 1 (PARP1), and heat shock protein-90 (Hsp90) [2, 11–13]. Through these methylations, SMYD2 is involved in several cellular processes including cell cycle progression, apoptosis, cellular differentiation, DNA damage response, and epigenetic gene regulation [2].

The crystal structures revealed that SMYD proteins have a bilobal structure [2, 4–7]. The N-lobe contains the SET, MYND, SET-I, and post-SET domains, and the C-lobe is made up of the CTD domain. The cofactor-binding site is located in a surface pocket in the N-lobe. The substrate-binding site is located between the N-lobe and CTD and situates at the bottom of a deep cleft. The orientation of the CTD is different among the SMYD family. This difference is reflected by the relative positions of the N- and C-lobes resulting in open and closed structures [6]. In SMYD2 the CTD is flexible and can undergo a conformational change upon binding to different cofactors [7]. Such a conformational change results in two SMYD2 structures with a slight difference in the size and shape of the substrate-binding pocket. The functional significance of the SMYD2 conformational change is still unknown. One possible consequence is that the conformational change may affect substrate access to the active site, thereby regulate substrate binding [5]. Another possibility is that the conformational change may be important for SMYD2 promiscuity allowing the structural adaptation to different substrates [5]. Finally, the conformational change may provide an allosteric mechanism for the effector-induced activity enhancement and change in substrate specificity [7].

Current understanding of the SMYD conformational change is limited to the structural differences observed in the crystal structures. The dynamical nature of the SMYD proteins is still poorly understood. It remains unknown of the structure of dynamical networks and the pattern of correlated domain motions, both of which are fundamental in mediating substrate recognition and allostery [14, 15]. Using the molecular dynamics (MD) simulation, this study reveals that SMYD2 exhibits a negative correlated inter-lobe dynamics. Dynamical network analysis suggests optimal and suboptimal paths for such a correlation. This study provides insight into SMYD2 dynamics and could prove valuable in understanding SMYD2 substrate specificity.

## Materials and Methods

### Molecular Dynamics Simulation

Molecular dynamics simulation was performed using NAMD [16]. CHARMM force field was used to parameterize the simulation. Initial structure for simulation is the crystal structure of the SMYD2–SAH complex (PDB code: 3QWV; SAH: S-adenosyl-L-homocysteine or AdoHcy). The missing residues of the structure including two N-terminal residues and one C-terminal residue were filled using SWISS-MODEL [17]. The system was solvated inside an orthorhombic box of water molecules with a 13 Å padding in each direction. The system was then neutralized with NaCl at a concentration of 0.15 M. The final system contained 78,008 atoms. Simulation was performed with a 1 fs time step. Particle Mesh Ewald was used to treat long-range electrostatic interactions and a cutoff of 12 Å was used for non-bonded interactions. Periodic Boundary Conditions were applied during the simulation. The simulation started with 2,000 steps of energy minimization. The first half of the minimization had harmonic restraints on the protein, and the second half unrestrained minimization. The minimized structure was then slow heated from 0 to 300 K over 300 ps. At each integration step velocities were reassigned and the temperature was incremented by 0.001 K. The heated structure was then equilibrated for 300 ps and velocities were rescaled to 300 K at every integration step. The production run was performed in the NVE (microcanonical) ensemble at 300 K. The total simulation time was 2 ns and coordinates were recorded every 1 ps.

### Principal Component Analysis and Clustering

Principal component (PC) analysis was performed using Bio3D [18]. The entire 2 ns trajectory of 2000 frames was used in the analysis. The overall translational and rotational motions in the trajectory were eliminated by least squares fitting to the first frame. A 3 N × 3 N covariance matrix were generated using Cartesian coordinates of Cα atoms. Diagonalization of the covariance matrix generated 3 N eigenvectors, each having a corresponding eigenvalue. The trajectory was projected onto a particular eigenvector to reveal concerted motions. Clustering of the trajectory in the PC space was performed using *k*-means and hierarchical clustering algorithms. *k*-means partitions the observations into *k* clusters by minimizing the mean squared distance from each observation to its nearest cluster center. Hierarchical clustering builds a hierarchy of clusters based on the distance between the observations.

### Dynamical Network Analysis

Dynamical network analysis was done in VMD according to previous protocols [19, 20]. Each amino acid in the network was represented by one node and SAH by three nodes. Amino acid nodes were centered on Cα atoms and SAH nodes were located at atoms Cα, C4’, and N9. The edges between nodes were drawn if the residues were within a cutoff distance of 4.5 Å for at least 75% of the trajectory. The edge distances were derived from pairwise correlations which define the probability of information transfer across the edge. Correlations were calculated from the trajectory by the program Carma [21]. The community substructure of the network was obtained using the Girvan-Newman algorithm. Nodes in a community have more and stronger connections within that community than the nodes in other communities. Critical nodes were defined based on the betweenness, which measures the importance of a node to the entire network. Critical nodes connect communities and lie at the interface between pairs of communities. Optimal and suboptimal paths were generated from the initial dynamical network matrix. The optimal path is the shortest path between two given nodes. Suboptimal paths are paths that are slightly longer than the optimal path.

## Results

### 1. Dynamical Details of SMYD2–SAH Complex Structure

**Fig 1:**
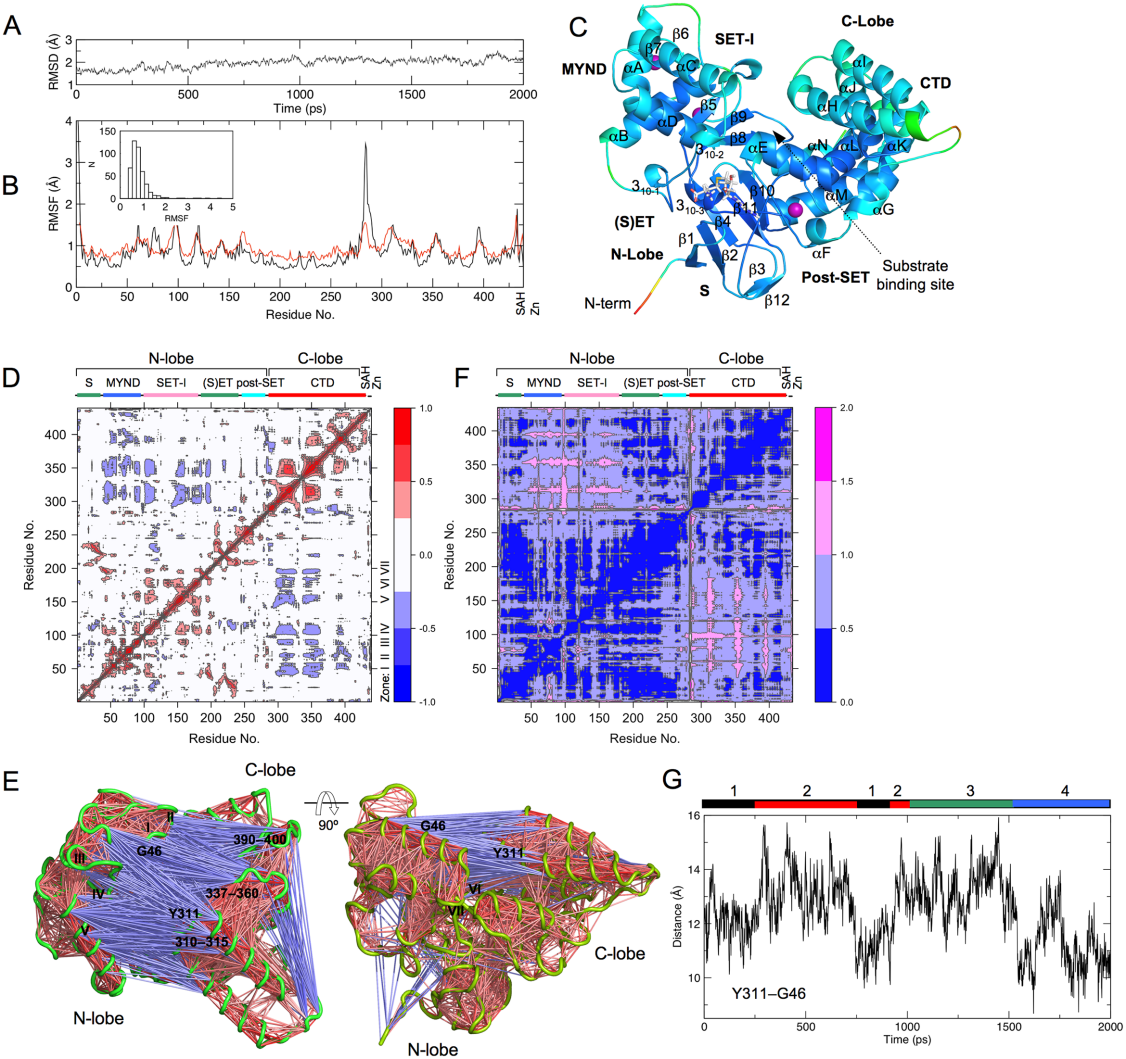
SMYD2 dynamics. (A) Backbone RMSD during the simulation. RMSD was calculated relative to the crystal structure. (B) Root mean square fluctuation (RMSF) of Cα atoms during the simulation (black line). Red line depicts the RMSF values converted from crystallographic B-factors. The inset depicts the distribution of the simulation RMSF. (C) Ribbon diagram of SMYD2 structure at 2 ns. The structure is colored according to the simulation RMSF. Color scale from blue to red depicts low to high atomic fluctuations. Secondary structures, α-helices and β-strands are labeled and numbered according to their position in the sequence. SAH is represented by sticks and zinc ions by purple spheres. (D) Cross-correlation map of the trajectory. Blue indicates a negative correlation between residue fluctuations, and red depicts a positive correlation. Lobe and domain structures of SMYD2 are indicated on the top of the map. (E) Visualization of residue–residue cross-correlations. SMYD2 is depicted by green coils. Blue and red lines indicate negative and positive correlated motions. (F) Inter-residue distance deviation map. Color scale from blue to magenta depicts small to large distance deviations. (G) Distance fluctuation of Y311–G46 during the simulation. Color bars depict the conformer clustering results obtained in Fig 2.

The simulation was performed using NAMD [16]. The starting structure is the crystal structure of SMYD2–SAH complex. The simulation was done in the NVE ensemble. The system was slow heated and equilibrated before 2 ns production simulation. The stability of the system during the production stage was evident by stable kinetic energy, potential energy, temperature, and RMSD (root mean square deviation) (data in S1 Fig and Fig 1A). The protein structure does not significantly deviate from the crystal structure. The backbone RMSD fluctuates between 1.4 Å and 2.5 Å with an average value of 2.0 Å.

SMYD2 dynamics revealed by the MD is similar to that from the crystallographic B factor-based analysis (Fig 1B and 1C). Most of the residues have a below 1 Å atomic displacement. The least dynamical region is the SET domain. The SET is the catalytic domain responsible for cofactor binding and substrate binding. The post-SET which is tethered to the SET by a zinc ion also shows a less dynamical structure. The most dynamical region in SMYD2 is found in the N-termini. In the crystal structure the first two N-terminal residues were not observed. The second largest displacement is found at the linker region between the post-SET and CTD. This non-conserved region has a variable length in SMYD proteins. This region has been proposed to act as a hinge for inter-domain movement [6, 7]. Large motion is also observed for parts of the MYND and CTD. In CTD, the most dynamical regions are the linker regions between the pairs of up-down helices. In MYND, the variable regions are the N- and C-terminus of the kinked helix αA and a loop between β5 and β6. In SET-I, the most dynamical regions are the end of the first helix (αB) and the beginning and end of the second helix (αC), and the loop forming the cofactor-binding site is relatively static.

### 2. Correlated Inter-Lobe Motion

The CTD and N-lobe show strong negative correlated dynamics (Fig 1D). The regions in the CTD involved in such a correlation include residues 300–315, 337–360, and 390–400. These regions are among the most dynamical regions in the structure (Fig 1B) and located at the inner surface of the C-lobe (Fig 1E). The correlated regions in the N-lobe are divided into seven zones spanning from residues 40–200. The zones include the following regions: (I) residues 41–53; (II) 68–73; (III) 85–95; (IV) 100–115; (V) 150–160; (VI) 183–185; (VII) 195–200. These zones spread across the MYND, SET-I and part of the SET domain but are clustered at the inner face of the N-lobe. As a result, the two correlated sets of residues are facing each other across the gap of the N- and C-lobes (Fig 1E). The residues in each set show positive correlated intra-lobe dynamics, whereas the two sets are related by the negative correlated inter-lobe dynamics (Fig 1D and 1E). During the simulation the contact distances between the residue-pairs of the two sets vary significantly (Fig 1F). The level of variation is two times above the average variation. This indicates a relatively large distance variability between the N- and C-lobes. This together with the negative correlated inter-lobe dynamics suggests a possible clamshell-like motion or open–closed motion between the lobes. The distances of the two representative residues, Y311 in the C-lobe and G46 in the N-lobe, range from 8.7 to 16.0 Å during the simulation. The fluctuated pattern of the distance indicates a slightly open and closed conformation (Fig 1G).

### 3. Principal Component Analysis

**Fig 2:**
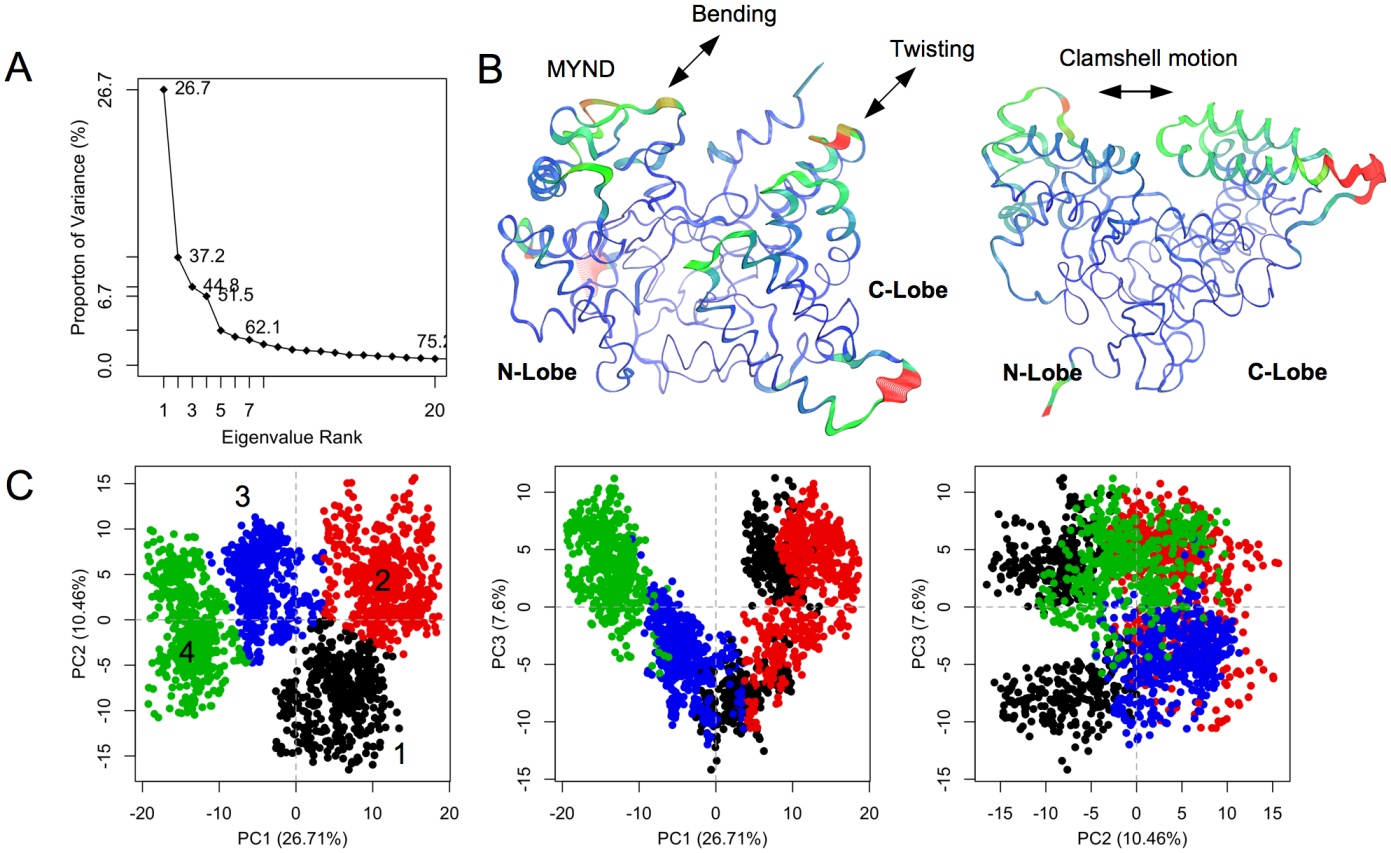
Principle component analysis. (A) Scree plot showing the proportion of variance against its eigenvalue rank. (B) Visualization of the motions along PC1 (left) and PC2 (right). The most dissimilar structures along a given PC are depicted by thicker coils. The interpolated structures produced by Bio3D [18] are shown by thinner coils. Color scale from blue, green, to red depicts low to high atomic displacements. (C) Projection of the trajectory onto the planes formed by the first three principle components. Conformers are colored according to the *k*-means clustering: cluster 1, black; 2, red; 3, blue; 4, green.

To further understand SMYD2 correlated dynamics, principal component (PC) analysis was performed using Cα position covariance (Fig 2A). The first PC accounts for more than one fourth of the overall variance. The second PC accounts for 10%. The first three components together account for 45%. The individual component contributions afterward drop below 6%. The first PC describes a twisting motion of the CTD with respect to the N-lobe and a spring-bending motion within the MYND (Fig 2B). The second PC is dominated by a clamshell-like motion between the N- and C-lobes. It is therefore that the variance in the PC1–PC2 plane essentially dictates the negative correlated inter-lobe dynamics. Based on these variances, the conformers throughout the simulation were grouped into four clusters using *k*-means algorithm (Fig 2C). The number of clusters was chosen based on the “elbow criteria”. At a cluster count of four the BSS/TSS (Between-group Sum of Squares/Total Sum of Squares) ratio is 82.8%. Similar clustering was obtained using hierarchical clustering algorithm (data in S2 Fig). Cluster 1 populates in the first 0.25 ns and between 0.7 and 0.9 ns. Cluster 2 is intertwined with Cluster 1 (0.25–0.7 ns and 0.9–1.0 ns). Cluster 3 is sampled in a time window of 1.0–1.5 ns. Cluster 4 lasts for the remainder of the simulation. This PC1–PC2 plane-based clustering appears to correlate with the pattern of distance fluctuation between Y311 and G46 (Fig 1G). The Y311–G46 distance represents the distance between the N- and C-lobes or the open/closed state of the structure. Cluster 1 and 4 correspond to the closed state, while cluster 2 and 3 sample the open one.

### 4. Dynamical Network Analysis

**Fig 3:**
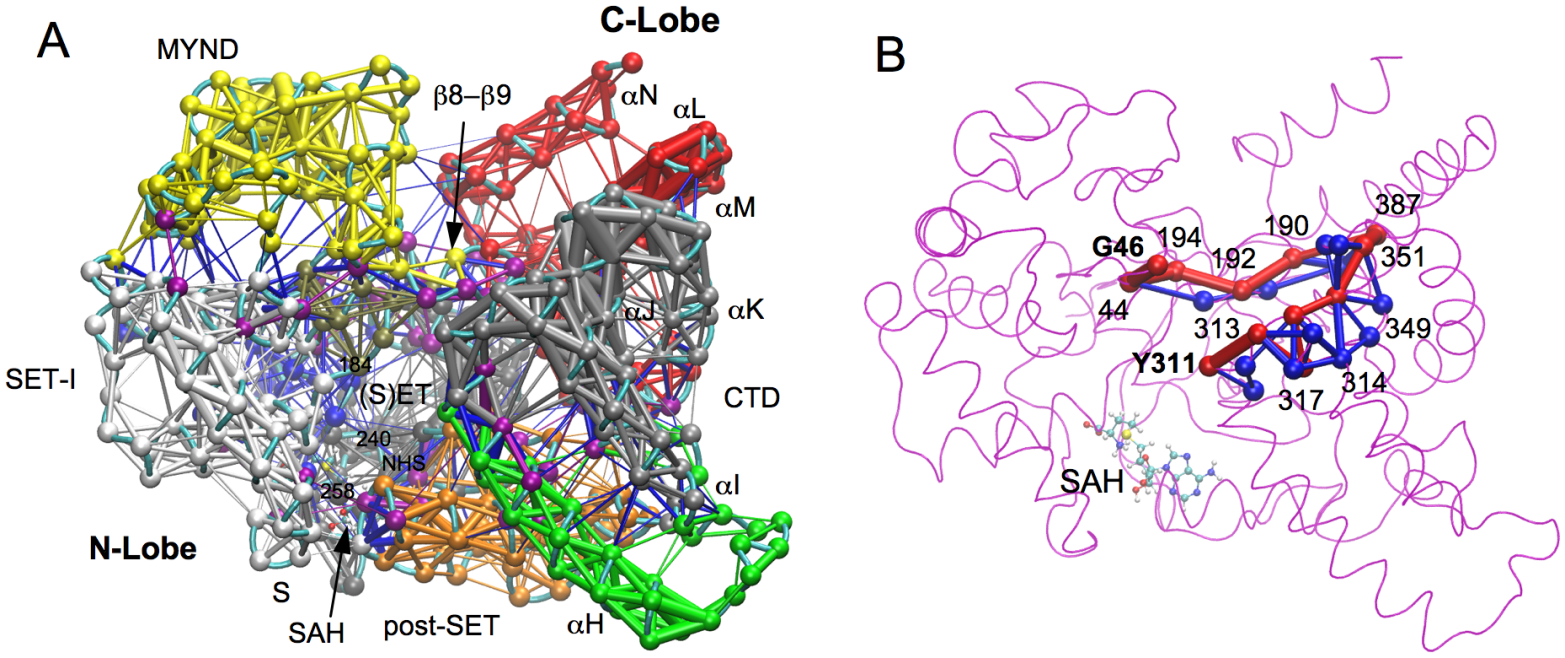
Dynamical network analysis. (A) SMYD2 dynamical network. The network is colored according to communities. Points in the network are nodes, and lines between the nodes represent edges. The thicker lines depict the stronger edges or stronger correlations. Critical nodes are colored in purple. (B) Optimal and suboptimal paths between Y311 and G46. The optimal path is colored in red and suboptimal paths in blue. The edge thickness is weighted by the number of suboptimal paths crossing the edge. Residues along the optimal path are labeled.

Dynamical network analysis was performed to define the allosteric paths for SMYD2 correlated inter-lobe dynamics. This analysis revealed nine communities in the dynamical structural network (Fig 3A). The community assignment is roughly correlated with the sequence- and structure-based domain assignment [4, 6]. The SET is split into two communities largely corresponding to the S-sequence and core SET. The cofactor product SAH is associated with the S-sequence community. This indicates a stronger correlated motion between SAH and the N-terminal S-sequence. The S-sequence has been shown to be involved in cofactor binding [4, 6, 7]. Mutation of two Gly residues in this sequence abolished SMYD1 enzymatic activity [4]. The SET-I, which is also involved in cofactor binding, forms a separate community. The MYND, a protein–protein interaction module, forms another community. There is a separate community formed at the interface of SET, MYND, and SET-I. This community contributes the residue Phe184 to the target lysine access channel. However, other two aromatic residues (Y240 and Y258) in this channel belong to the S-sequence-containing community. Most of residues in the post-SET belong to one community. This community also contains the residue H207 from the conserved active site motif NHS. H207 chelates the zinc atom of the post-SET, which may result in such an association. Other two residues (N206 and S208) in the NHS motif belong to the S-sequence-containing community. In CTD there are three communities formed by the first helix (αH), αI–αJ–αK, and αL–αM–αN. The predicted Hsp90 binding site is located between the second and third communities, which is also the extended ribosomal binding site [2, 5, 7].

The communication between network communities is mediated through critical nodes [20]. Such nodes are important for allosteric signal transduction and dynamical correlation between the communities [20, 22]. Of note, the β8–β9 hairpin contains four critical nodes (residues 190–193). These nodes form a bridge connecting the N- and C-lobes (Fig 3A). Two of these nodes (residues 190 and 191) have direct interaction with the CTD. Disrupting this interaction has been found to reduce SMYD2 methyltransferase activity [23]. This suggests that the β8–β9 hairpin may represent an optimal path for dynamical inter-lobe communication. The optimal and suboptimal paths were generated between Y311 and G46. As mentioned earlier, these pair of residues move in concert toward the opposite direction. Their dynamical relationship can represent the open and closed state of the structure and correlated inter-lobe dynamics. The optimal path between the two residues passes through the β8–β9 hairpin (Fig 3B). All suboptimal paths also pass through the hairpin. The β8–β9 hairpin occurring in the highest number of suboptimal pathways may thus be necessary to guarantee an effective pathway for inter-lobe communication.

## Discussion

The crystal structures revealed that SMYD proteins adopt different CTD conformations [2, 4–7]. SMYD1 has an open CTD structure with the substrate-binding cleft completely exposed. SMYD3 has the narrowest substrate-binding cleft due to the direct CTD–N-lobe interaction [6]. SMYD2 is like a conformational intermediate, and when different cofactors bound, the CTD exhibits different conformations [7]. These data have suggested the dynamical nature of the CTD and a possible open–closed motion of the two lobes [2]. Our MD simulation of SMYD2 structure supports an open–closed motion. The simulation reveals that SMYD2 exhibits a negative correlated inter-lobe dynamics, and this correlated dynamics is described by a twisting motion of the CTD with respect to the N-lobe and a clamshell-like motion between the lobes. Correlated inter-domain motions may mediate fundamental protein functions such as substrate recognition [15]. In SMYD2 the substrates bind to the protein in a U-shaped conformation [5, 23]. Both the N- and C-lobe contribute to the binding. The inter-lobe dynamics will alter the size of the substrate-binding site. The coupling of the two lobes by the correlated motion might offer the specificity and promiscuity for substrate recognition. Correlated inter-domain motions are also important for allostery [14]. In SMYD2 the cofactors exhibit allosteric effects. Binding of sinefungin and SAH to the cofactor-binding site in the N-lobe caused a structural difference in the CTD [7]. Such a long-range structural effect could not be explained by the crystallographic studies [7], but the correlated inter-lobe dynamics might provide a signal transduction pathway enabling a long-range domain–domain communication.

A complex mechanism regulates SMYD biochemical function. Binding of Hsp90 to the CTD significantly enhances the activity of SMYD proteins [3, 24, 25]. For SMYD2, Hsp90 binding not only increases the activity but also changes the substrate specificity [25]. Both SMYD2 and SMYD3 interact with DNA [3, 26]. DNA binding to the MYND has been shown to enhance SMYD3 enzymatic activity [27]. The mechanism of such an activity enhancement is unknown, but one possible mechanism is that the binding may affect the domain dynamics and inter-lobe dynamical correlation. Such an effect could be transduced to other parts of the protein through the critical nodes bridging the communities, which in turn might impact substrate binding and cofactor binding.

Studying SMYD2 conformational dynamics is of therapeutic interest. Dynamical information of SMYD2 structure would facilitate receptor-flexibility-enabled drug design. The conformational states sampled by the MD simulation can be used in ensemble docking. In addition, the identification of the critical nodes and optimal path mediating the dynamical network communication could offer new strategies to manipulate SMYD2 function. Disrupting a specific network communication could represent a rational approach for the design of drugs with improved potency and selectivity. In summary, the MD simulation of SMYD2 structure has revealed that SMYD2 exhibits a negative correlated inter-lobe dynamics and provided additional insight into the structure of this multifunctional protein lysine methyltransferase.

## Supporting Information

S1 Fig System stability during the production simulation. Kinetic energy (*E*_k_), potential energy (*E*_p_), and temperature were plotted as a function of time.
(TIF)

S2 Fig Trajectory clustering in PC1–PC2 subspace. (A) BSS/TSS ratio against the *k*-means cluster count. (B) A dendrogram obtained using a complete-link hierarchical clustering algorithm. Color bars depict the clustering results at a cluster count of four. (C) Projection of the trajectory onto the planes formed by the first three principle components. Conformers are colored according to the hierarchical clustering: cluster 1, black; 2, red; 3, blue; 4, green.
(TIF)
