# Supplementary figures and images for: Molecular Dynamics Simulation Reveals Correlated Inter-Lobe Motion in Protein Lysine Methyltransferase SMYD2
Source: PLoS One. 2015 Dec 30;10(12):e0145758. doi: 10.1371/journal.pone.0145758 (PMC4696779; doi:10.1371/journal.pone.0145758)

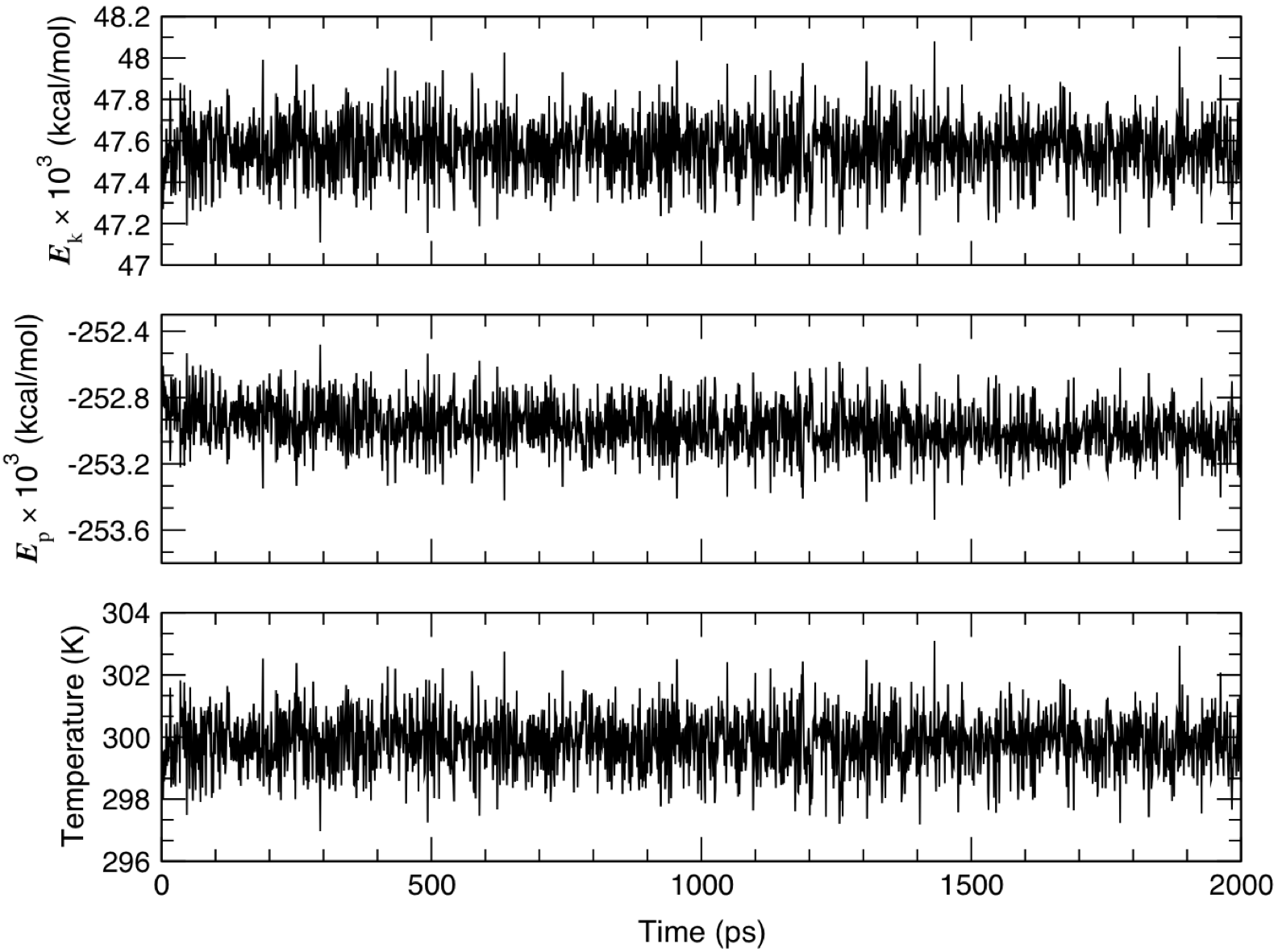

Supplement: S1 Fig — Kinetic energy (E k), potential energy (E p), and temperature were plotted as a function of time. (TIF) [file pone.0145758.s001.tif]

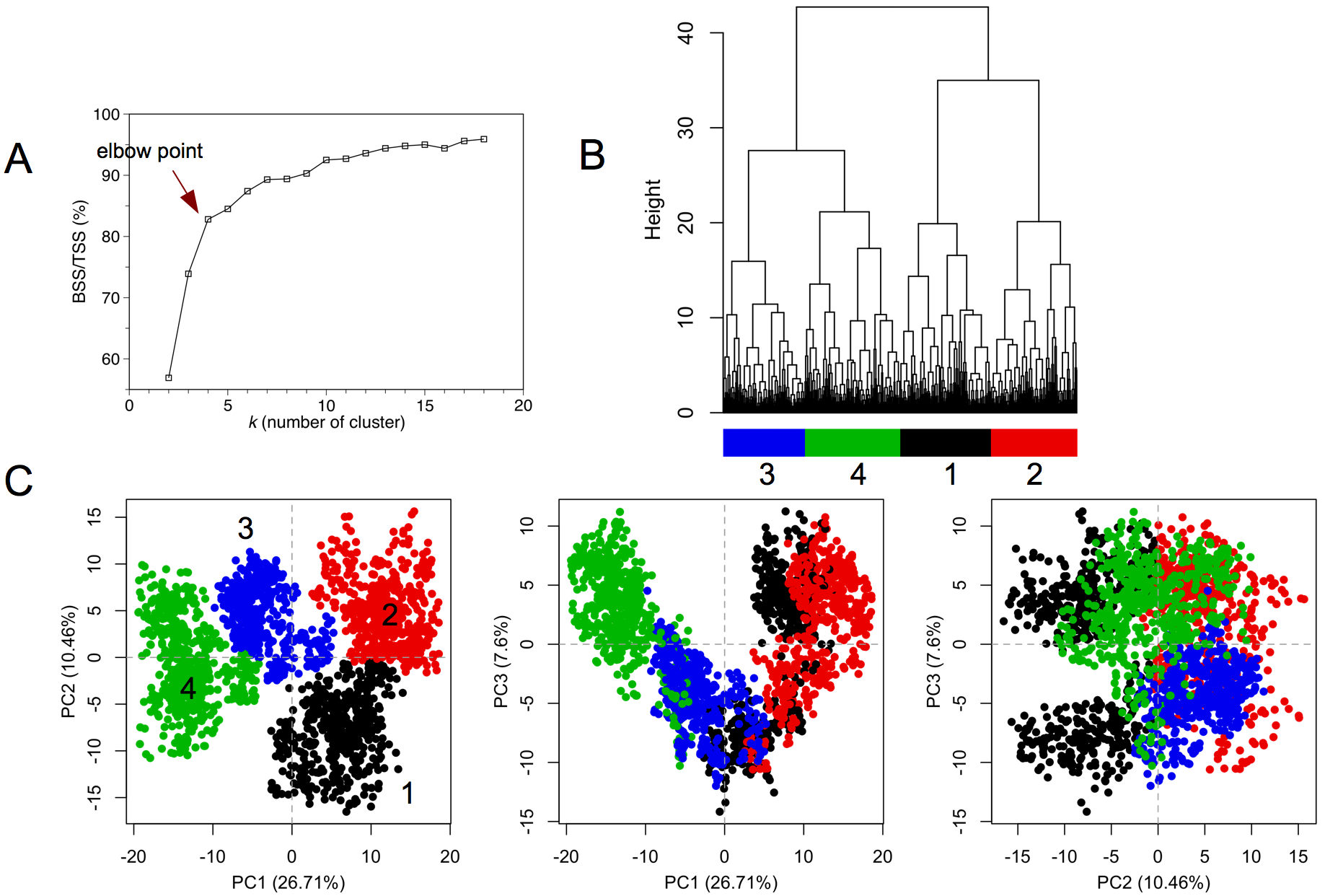

Supplement: S2 Fig — (A) BSS/TSS ratio against the k-means cluster count. (B) A dendrogram obtained using a complete-link hierarchical clustering algorithm. Color bars depict the clustering results at a cluster count of four. (C) Projection of the trajectory onto the planes formed by the first three principle components. Conformers are colored according to the hierarchical clustering: cluster 1, black; 2, red; 3, blue; 4, green. (TIF) [file pone.0145758.s002.tif]
